# Detection of Stress in Cotton (*Gossypium hirsutum* L.) Caused by Aphids Using Leaf Level Hyperspectral Measurements

**DOI:** 10.3390/s18092798

**Published:** 2018-08-24

**Authors:** Tingting Chen, Ruier Zeng, Wenxuan Guo, Xueying Hou, Yubin Lan, Lei Zhang

**Affiliations:** 1 College of Agriculture, South China Agricultural University, Guangzhou 510642, China; chentingting@scau.edu.cn (T.C.); ruierzeng@126.com (R.Z.); h_xy123@126.com (X.H.); 2 Department of Plant and Soil Science, Texas Tech University, Lubbock, TX 79409, USA; wenxuan.guo@ttu.edu; 3 College of Engineering, South China Agricultural University, Guangzhou 510642, China

**Keywords:** cotton, aphid, remote sensing, biotic stress detection, hyperspectral indices

## Abstract

Remote sensing can be a rapid, accurate, and simple method for assessing pest damage on plants. The objectives of this study were to identify spectral wavelengths sensitive to cotton aphid infestation. Then, the normalized difference spectral indices (NDSI) and ratio spectral indices (RSI) based on the leaf spectrum were obtained within 350–2500 nm, and their correlation with infestation were qualified. The results showed that leaf spectral reflectance decreased in the visible range (350–700 nm) and the near-infrared range (NIR, 700–1300 nm) as aphid damage severity increased, and significant differences were found in blue, green, red, NIR and short-wave infrared (SWIR) band regions between different grades of aphid damage severity. Decrease in Chlorophyll a (Chl a) pigment was more significant than that in Chlorophyll (Chl b) in the infested plants and the Chl a/b ratio showed a decreasing trend with increase in aphid damage severity. The sensitive spectral bands were mainly within NIR and SWIR ranges. The best spectral indices NDSI (R_678_, R_1471_) and RSI (R_1975_, R_1904_) were formulated with these sensitive spectral regions through reducing precise sampling method. These new indices along with 16 other stress related indices compiled from literature were further tested for their ability to detect aphid damage severity. The two indices in this study showed significantly higher coefficients of determination (R^2^ of 0.81 and 0.81, *p* < 0.01) and the least RMSE values (RMSE of 0.50 and 0.49), and hence have potential application in assessing aphid infestation severity in cotton.

## 1. Introduction

Cotton is an important commercial crop grown in about 70 countries for fiber, and an estimated 20 million tons of cotton is produced each year in the world. However, pest and diseases are some of the major constraints in cotton production, which cause reduced yields every year, posing a significant risk to cotton production worldwide [1]. In recent years, following the introduction of Bt cotton, farmers in China and elsewhere have faced challenges of rising incidence of sap feeders such as aphids, mealybug, and leafhoppers [2]. The cotton aphid is generally viviparous in the growing season and grows quickly to adulthood and populations rapidly increase [3]. Aphid infestation causes damage through piercing and sucking leaves as well as transmitting virus. Infested leaves often cup downward and appear crinkled. Heavy infestations on some crops may decrease photosynthesis activity and can cause stunting of young plants and considerably reduce cotton yields [4]. Cotton aphid control is primarily dependent on the application of insecticides, often with multiple applications in each season in China. However, misuse and overuse of fungicide/insecticide could result in failure of disease/insect control, and even soil contamination [5]. In addition, an inaccurate application of insecticide can lead to missing infected areas or overuse, especially when using automatic spray system. To more effectively and efficiently control aphid infestation, it is important in practice to assess the spatial distribution of its infestation in time to guide the spray of insecticide. 

The most common and conventional way of assessing aphid infestation is through field survey. The traditional ground-based survey method, however, is expensive and inefficient, especially over large areas. Remote sensing may be a possible alternative for obtaining the spatial distribution information of aphid over a large area with a relatively low cost [6]. Use of remote sensing techniques for such purpose is based on the assumption that stresses induced by aphid infestation affects photosynthesis and physical structure of the plant, resulting in the alteration of light absorption and plant reflectance characteristics [7]. As plant stress may be characterized using specific responses in the visible, near infrared and shortwave infrared spectral domains, it is possible to detect or map plant response to insects with remotely sensed data [8]. Recent development in optical technology has made it possible to differentiate diseases and healthy crop, and thus the prospect of automatically measuring the spatial distribution of crop disease and insect pests.

Many studies have been undertaken to characterize reflectance spectra of crop damage due to pests such as leafhopper [9], solenopsis mealybug [10] and especially aphid [11] worldwide. To extract information of target objects, many methods have been adopted, including vegetation parameters based on sensitive wavelength, regression analysis and radiative transfer modeling. These approaches attempt to minimize background noise and to enhance the capacity of spectral information utilization and the accuracy of the estimating models [12,13,14,15]. Among them, the normalized difference vegetation index (NDVI) and ratio vegetation index (RVI) have been widely used to analyze multi-spectral information in crop plants, because they are constructed in simple form for easy calculation [16,17]. However, due to the lack of effective means to analyze hyperspectral information within the full range of 350–2500 nm wavelengths [18], it is challenging to systematically determine hyperspectral indices that might provide valuable information indicating the biochemical components in plants. In addition, very few studies have examined the relationship between leaf spectral reflectance and the damage severity grades caused by aphid for detection of aphid damage. Therefore, further investigations are needed to explore and identify novel sensitive bands from hyperspectral data, to develop simpler vegetation indices (NDSI and RSI), and to develop monitoring models that are more accurate and have wider applicability in estimating pest damage based on hyperspectral sensing information. Hence, this study was undertaken with the following objectives: (i) investigate the effect of aphid infestation on chlorophyll pigments and relative water content in cotton; (ii) characterize spectral signatures from cotton leaf infested with aphid at the leaf level; and (iii) identify spectral bands sensitive to cotton aphid damage, derive hyperspectral vegetation indices and develop models for damage assessment at the leaf level. 

## 2. Materials and Methods

### 2.1. Field Site and Sampling

This study was conducted in the Anyang district of Henan Province, China during the 2017 season. Two cotton fields (Table 1) with a natural, but widespread and severe outbreak of aphid were identified for the study. The crop was grown under recommended regimens of fertilizer application and irrigation. The crop was free from stresses other than the target pest, and no pesticide was applied until after data collection in the two fields. Based on the visible appearance of symptoms, each selected plant was categorized into one of the five grades of aphid damage severity, from Grade 0 (healthy) and Grade 1 (low infestation) to Grade 4 (severe damage) (Table 2). About 10–12 cotton leaves under each Grade were selected form each location, depending in their availability at the time of recording observations. A total of 86 cotton leaf spectra were sampled across the two fields.

### 2.2. Data Collection

#### 2.2.1. Leaf Spectra Data Measurement

Leaf reflectance data were collected with a FieldSpec 3 Hi-Res spectroradiometer (ASD Inc., Boulder, CO, USA; spectral range: 350–2500 nm). The sampling interval was 1.4 nm at 350–1000 nm range, and 2 nm at 1000–2500 nm range. The leaves were collected and immediately sealed in plastic bags, kept in an ice chest, and then transported to the laboratory for spectral measurements. The measurements were conducted followed those described by Pu et al. (2003) [20]. The light source was a 100 W halogen reflectorized lamp. All spectra were measured at the nadir direction of the radiometer at a 25° field of view (FOV). A standard whiteboard was used as white reference and then measured every five minutes to convert leaf radiance to spectral reflectance. The reflectance spectra of the leaves, randomly picked from the upper hemisphere of the leaves, were collected by measuring 10 mm-diameter spots using a plant probe. Each leaf sample consisted of an overlapping pile of 2–3 leaves to eliminate possible background effect (black cloth) on the spectrum (based on our experiment, the spectrum of an overlapping pile of two cotton leaves becomes stable). The adaxial surfaces of a sample were measured five times, from which an average spectral reflectance curve was generated. Resultant data were interpolated using the ASD ViewSpecpro software for post-processing to produce values at 1 nm interval.

#### 2.2.2. Estimation of Leaf Chlorophyll Concentration

After measuring the reflectance from selected plants, one leaf disc of 20 mm diameter was punched from the uppermost fully expanded leaf of each plant, and immediately placed in a vial with 15 mL dimethyl sulfoxide (DMSO) for chlorophyll extraction. The vials containing leaf samples were incubated at room temperature in the dark for 24 h to allow for complete extraction of chlorophyll into the solution. After centrifugation for 10 min at 3500× *g*, the absorbance of the supernatant was measured at 663 and 646 nm with a UV-VIs spectrophotometer (T6, Shanghai, China). Levels of chlorophyll were measured using the method described by Wellburn (2004) [21].

#### 2.2.3. Estimation of Relative Water Content (RWC)

After collection of leaf disc samples for chlorophyll, the remaining leaf sample was kept in sealed polythene covers in an ice box and transferred to a laboratory for further analysis. Each sample was immediately weighed (Fresh weight, FW), and was cut into small pieces and soaked in water for 4 h for measuring turgid weight (TW). Thereafter, the samples were dried in an oven at 80 °C until a constant weight (dry weight, DW) was reached. RWC was calculated as described by Smart and Bingham [22].

RWC = [(FW − DW)/(TW − DW)] × 100(1)

### 2.3. Data Analysis

The raw DN values recorded from the field were converted to reflectance values using the ASD ViewSpecPro software [23]. Spectral data from healthy and severely infested plants in the field across the spectral domain were initially averaged to five broad bands: blue (450–520 nm), green (521–600 nm), red (630–690 nm), near infrared (760–900 nm), and mid infrared (1550–1750 nm). These five broad bands correspond to the LANDSAT Thematic Mapper (TM) spectral bands 1–5. Paired *t*-test was performed on the mean reflectance data from healthy and infested plants in these five bands [24]. Person correlation coefficient (r) between the aphid infestation and the reflectance at each 1 nm wavelength was calculated from the data of Field 1 and Field 2 (*n* = 86), and correlation intensity curves were plotted to assess the relationship between them. 

In this study, all possible two-band combinations of spectral indices (NDSI and RSI) ranging from 350 to 2500 nm were constructed in the form of a matrix linkage [25]. RSI and NDSI were used based on the following corresponding equations:

(2) $$\mathrm{RSI}=\frac{R_{\lambda 1\;}}{R_{\lambda 2}}$$

(3) $$\mathrm{NDSI}=\frac{R_{\lambda 1\;}-R_{\lambda 2}}{R_{\lambda 1}+R_{\lambda 2}}$$

where *R_λ_*_1_ and *R_λ_*_2_ are the spectral reflectance of random wavebands (*λ*1 and *λ*2, respectively). 

The new indices were developed using the independent data collected in Field 1 (*n* = 46). The new indices derived in our study along with 16 other choices compiled from the literature (Table 3) were subjected to linear regression analysis to quantify their relationship with aphid severity using the data in Field 2 (*n* = 40). Significant effects of aphid damage on leaf chlorophyll concentration and relative water content were determined by analysis of variance (ANOVA). Statistical significance of regression models and paired *t*-test was evaluated at *p* = 0.05 and 0.01 for the whole dataset. All these procedures for the statistical analysis and contour mapping of the R^2^ and SE values were completed using a MATLAB (Mathworks, 2000) script. The root mean square error (RMSE), R^2^ and the slope were used to evaluate the goodness of fit between the predicted and observed values. 

## 3. Result

**Figure 1:**
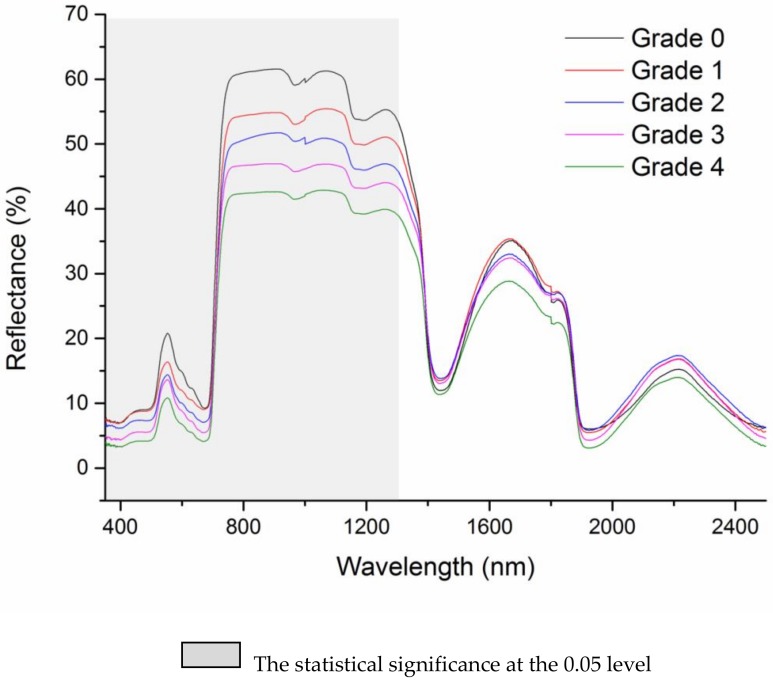
Mean reflectance spectra of cotton plants under different grades of aphid infestation.

The mean reflectance spectra of the 86 samples showed that the spectral response of the cotton leaves was affected by aphid infestation (Figure 1). With increasing grade of aphid damage, the leaf spectral reflectance decreased in the visible range (350 ≠ 700 nm) and in the NIR range (700–1300 nm). The paired *t*-test comparison of healthy and aphid infested plants revealed that the reflectance was significantly different among the blue, green, red, NIR and SWIR band region when the plants were in varying grade of aphid damage severity (Table 4).

### 3.1. Effect of Aphid Infestation on Chlorophyll and RWC

Spectrophotometric estimation of chlorophyll from healthy and aphid infested plants showed a significant decrease in Chl a, Chl b and RWC due to aphid infestation (Table 5). The total reduction in chlorophyll content (a + b) was between 14.83% and 65.39% for Grades 1–4 due to aphid infestation. There was no significant difference in RWC between Grade 0 and Grade 1. However, with increasing grade of aphid damage severity beyond Grade 1, a significant reduction in RWC in the affected plants was observed.

### 3.2. Identification of Sensitive Bands and Band Ratios

**Figure 2:**
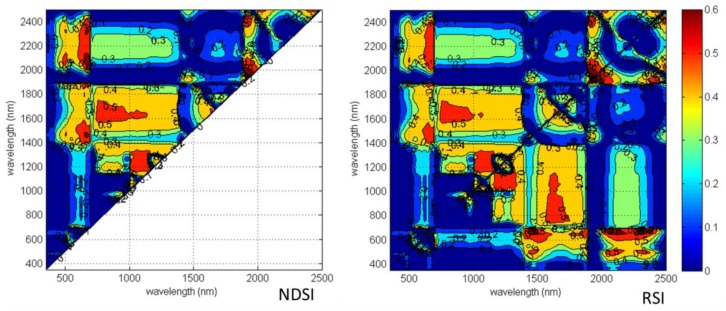
Contour maps of coefficients of determination (R^2^) for linear relationship between NDSI, RSI and aphid infestation grades of cotton leaves in 10 nm sampling interval at 350–2500 nm.

For systematic quantification of all possible two-band combinations of hyperspectral indices within the full range of 350–2500 nm, the reduced sampling method was adopted [25] to evaluate the relationship of aphid stress index (ASI) with NDSI and RSI from 350 nm to 2500 nm at 10 nm intervals based on data sets from Field 2 and to identify the sensitive band ranges with greater R^2^ values. Several “hotspots” with high correlation coefficients between ASI and NDSI or RSI were in the NIR and SWIR bands (Figure 2). As shown in the R^2^ contour map within the full wavelength, the R^2^ values are mostly greater than 0.55 using linear regression based on the NDSI of 600–1100 nm and 1400–2000 nm, and the RSI of 1850–2100 nm for ASI. 

**Figure 3:**
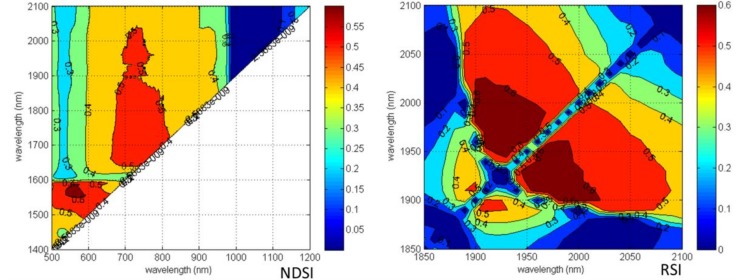
Contour maps of coefficients of determination (R^2^) for linear relationship between NDSI, RSI and aphid infestation grades of cotton leaves in 1 nm sampling interval at sensitive bands.

**Figure 4:**
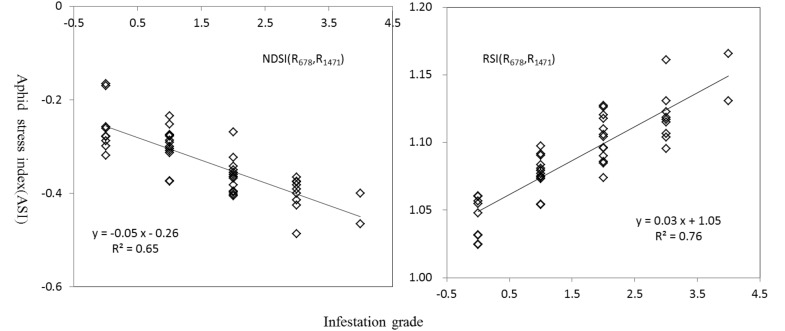
Regression of aphid infestation grades with the aphid stress indices.

Furthermore, through precise sampling of these sensitive spectral regions, more detailed contour maps of R^2^ values between ASI and NDSI or RSI at 1 nm intervals were obtained (Figure 3). Based on R^2^ values, the best cotton aphid stress indices among the selected NDSI and RSI groups were NDSI (R_678_, R_1471_) (R^2^ = 0.65) and RSI (R_1975_, R_1904_) (R^2^ = 0.76) for ASI, respectively (Figure 4).

### 3.3. Testing of Hyperspectral VIs for Aphid Damage Severity

The two new spectral vegetation indices developed using the reduced sampling method were further tested along with 16 other narrowband hyperspectral indices specific to crop stress complied from the literature for their predictability of aphid damage severity grades from sampled plants across Field 1 using linear regression analysis by setting aphid severity grade as dependent variable and spectral indices as independent variables. Three statistical parameters, namely, R^2^, RMSE, and slope between the observed and estimated values, were used to evaluate the performance of the established models comprehensively. The higher R^2^ values and the least RMSE values for ASI-1 and 2 (formulated using the reduced sampling method) showed that they are superior to all 16 other indices chosen from the literature (Table 6). However, plant pigment index (R^2^ = 0.02), two nitrogen indices (NSI-1 and NSI-2) (R^2^ range 0.02–0.05) and three Chl related indices (Chl SI 1–3) (R^2^ = 0.08) which were specifically developed for the cotton crop did not perform well for distinguishing aphid damage severity.

The monitoring models derived from NDSI (R_678_, R_1471_) and RSI (R_1975_, R_1904_) for ASI provided good accuracy, with R^2^ of 0.81 and 0.80, RMSE of 0.50 and 0.51, and slopes of 10.993 and 25.987, respectively. Overall, the validation results of the ASI monitoring models indicate good agreement between the estimated and observed values at different aphid damage severity in this study. 

## 4. Discussion

The spectral response of aphid damage on cotton was first observed by Reisig and Godfrey (2006). In their study, they found that wavelengths in the NIR were fair to moderately accurate predictors of aphid-infested cotton using aerial and satellite remote sensing data. However, to measure different severity degrees of aphid on cotton leaves from different developing stages of the pest would inevitably bring about a mixture phenomenon of phonological changes signals, with the aphid induced signals of the plant leaves [35]. To deal with this problem, our research attempts to detect the damage severity grades of a leaf directly based on the leaf reflectance, which could exclude this phenological impact. 

An independent two-sample *t*-test for broad-band comparison of reflectance from healthy and damaged plants with Grade 1 showed a significant difference in the blue, red, green, NIR and SWIR bands. Reflectance in the red bands was found significantly affected which indicated that aphid infestation affects the leaf chlorophyll and internal structure. Reduction in pigments and its resultant effect on reflectance spectra due to infestation by sap feeding insects such as cotton leafhopper [9] and rice brown plant hopper [36] has been reported. According to these studies, the change in leaf reflectance caused by pest damage is driven by the decrease of chlorophyll pigments and relative water content, as well as the breakdown of the cell structure. This process could lead to corresponding changes of reflectance in the visible and NIR regions, particularly around 470 and 670 nm [5]. In our research, it is obvious that the spectral reflectance in this region responded significantly to cotton aphid (Figure 1). 

Chlorophyll content is a potential indicator of vegetation stress because of its direct role in the photosynthesis process of light harvesting and initiation of electron transport [37]. It is interesting to note that in our study the decrease in Chl a pigment is significantly higher than Chl b. The decrease in chlorophyll a/b ratio was not significant in Russian wheat aphid. However, the ratio of Chl a/b showed a significant decreasing trend with increase in aphid infestation grades in our study, which is similar to the result of greenbug (*Schizaphis graminum Rondani*) damage in wheat [38]. Dai et al. (2009) observed that significant reduction in Chl a, Chl b and carotenoid contents were found in *Hypericum sampsonii* plant damaged by *thrips tabaci* [39]. Differences in reflectance between healthy and stressed vegetation due to changes in Chl a and b levels have been detected in the green peak and along the red-edge spectral region of 690–750 nm [37]. Therefore, the lower Chl a content in our study may partially explain the lower reflectance values in the red and blue regions.

Decrease in reflectance in NIR region could be attributed to the damage to leaf tissues by aphid. A breakdown in cell structure would lead to decreased internal scattering and increase in leaf transmittance in this region [40]. The sensitive wavelength at 1471 nm in this study could be attributed to water loss from the aphid infested cotton plants. This could be further corroborated with the significant R^2^ values observed between aphid infestation levels and RWC. Infestation of brown plant hopper in rice also gave rise to water stress symptoms and increased reflectance in SWIR region [36]. As shown in our study, the spectral changes in leaf reflectance caused by aphid damage is similar to those due to solenopsis mealybug in cotton [10].

Hyperspectral data provide leaf reflectance in large numbers of contiguous narrow bands. Analysis of large number of bands is complex and time consuming. In addition, a systematic quantification of hyperspectral indices on all possible two-band combinations ranging from 350 to 2500 nm is difficult to perform because of the lack of effective means to analyze hyperspectral information [18,41]. Therefore, development and optimization of band reduction techniques such as spectral vegetation indices using only a limited amount of data are important and useful in the detection of plant stress. The present study adopted a different method of reducing precise sampling to detect the aphid damage severity through spectroscopy technology to explore consistent feature bands and construct simpler spectral indices, and to develop more accurate models with wider applicability. 

Several studies have developed spectral indices that use sensitive bands for detection of crop damage due to solenopsis mealybug, leafhopper, and spider mites [9,10,42], previously. The developed indices are based on the most relevant wavelength and normalized wavelength combinations regarding to a plant pest. They are characterized by a high sensitivity and specificity for the detection and identification of the different pest of cotton. In this paper, we constructed new and simple spectral indices of NDSI (R_678_, R_1471_) and RSI (R_1975_, R_1904_) for ASI. The vegetation index (NDSI and RSI) composed of the NIR and SWIR bands should be able to well detect the damage severity grades in cotton plants. As a result, the models derived from this study are simple and applicable, with reasonable explanation from the principle of remote sensing. 

Some of the earlier developed stress related vegetation indices like Simple ratio, Disease water stress index, Leaf hopper index, mealybug stress index 1 and mealybug stress index 2 when tested in our study for aphid damage showed good result (R^2^ range 0.40–0.62), but not better than the newly developed ASI (R^2^ range 0.80–0.81). However, the indices developed earlier, specifically in cotton, for remote sensing of nitrogen stress [32,33] did not show better results when tested for aphid damage. The possible reason maybe that available N has a direct and proportionate impact on leaf chlorophyll content compared to water stress. However, in addition to reduction in chlorophyll, the aphid damage reduces relative water content in our study. Additionally, it caused changes in the leaf structure in the form of crinkling and twisting of leaves, stunted growth, of which such symptoms are not found with the nitrogen stress. Indices such as NDVI and LHI are standardized indices that use characteristic features of vegetation spectra, namely low reflectance in the red region of the spectrum due to chlorophyll absorption and high reflectance values in the NIR region due to scattering caused by internal leaf structure. 

Compared with the models based on the other 16 indices chosen from the literature, the performances of the ASI models based on these new sensitive spectral indices are improved, and showed strong relationship with the aphid severity. The superior performance of NDSI and RSI could be due to the fact that the reducing precise sampling method used in developing these indices and could be used for the detection of aphid damage severity grades in cotton plants. The present study provides evidence that spectral pest indices will improve and simplify plant pest detection based on hyperspectral data. This can enable effective planning and implementation of area-wide integrated pest management practices.

## 5. Conclusions

This study showed that aphid infestation significantly reduces chlorophyll and relative water content in cotton leaves. Broad band comparison of mean reflectance spectra between healthy and aphid infested plants showed significant decrease in the visible range (350–700 nm) and in the NIR range (700–1300 nm), which enables the detection of aphid infestation by remote sensing. A series of novel wavelengths for ASI were identified by the reducing precise sampling method. Two sets of new sensitive spectral indices NDSI (R_678_, R_1471_) and RSI (R_1975_, R_1904_) were determined and performed better than other narrow band indices compiled from the literature, and hence have potential use for detecting grades of aphid damage severity in cotton.

## Figures and Tables

**Table 1:** Details of the field locations selected and sampling adopted for the study.

	Field 1	Field 2
Latitude	36°08′ N	36°08′ N
Longitude	114°51′ E	114°49′ E
Altitude (m)	320	320
District	Anyang	Anyang
State	China	China
Field size (m^2^)	1200	1800
Cultivars	CCRI-79	CCRI-79
Date of sowing	10 April 2017	10 April 2017
Sampling date	24 May 2017	24 May 2017
Sampling time	10:30–12:30	10:30–12:30
Sampling number	46	40

**Table 2:** Grading of cotton plants infested by aphid.

Grade	Symptom of Aphid Infestation
0	Healthy plant with no pests
1	Few aphids scattered over the plant. Foliage free from crinkling or curling with no yellowing symptoms
2	Crinkling and curling of few leaves in the upper portion of plant
3	Crinkling and curling of leaves all most all over the plant
4	Extreme curling, crinkling and drying of leaves all over the plant, plant growth hampered

Source: Kranthi et al. (2009) [19] (modified).

**Table 3:** Vegetation indices used in this study.

	Spectral Vegetation Index	Formula	References
1	Simple Ratio (SR)	(R_695_/R_420_)	Carter (1994) [26]
2	Normalized Difference Vegetation Index (NDVI)	(R_800_ − R_670_)/(R_800_ + R_670_)	Rouse et al. (1974) [27]
3	Disease Water Stress Index 2 (DWSI-2)	(R_1660_/R_550_)	Apan et al. (2004) [28]
4	Aphid index (AI)	(R_761_ − R_908_)/(R_712_ − R_719_)	Mirik et al. (2006) [29]
5	Damage sensitive Spectral Index-2 (DSSI 2)	(R_747_ − R_901_ − R_537_ − R_572_)/(R_747_ − R_901_) + (R_537_ − R_572_)	Mirik et al. (2006) [30]
6	Chlorophyll Index (CI)	(R_415_ − R_435_)/(R_415_ + R_435_)	Barnes (1992) [31]
7	Chl Stress Index 1 (Chl SI-1)	(R_415_/R_695_)	Read et al. (2002) [32]
8	Chl Stress Index 2 (Chl SI-2)	(R_708_/R_915_)	Zhao et al. (2005) [33]
9	Chl Stress Index 3 (Chl SI-3)	(R_551_/R_915_)	Zhao et al. (2005) [33]
10	Leaf Hopper Index (LHI)	(R_761_ − R_691_)/(R_550_ − R_715_)	Prabhakar et al. (2011) [9]
11	Nitrogen Stress Index 1 (NSI-1)	(R_415_/R_710_)	Read et al. (2002) [32]
12	Nitrogen Stress Index 2 (NSI-2)	(R_517_/R_413_)	Zhao et al. (2005) [33]
13	Mealybug Stress Index-1 (MSI-1)	(R_550_ + R_768_ + R_1454_) − [R_1454_/(R_550_ + R_768_)]	Prabhakar et al. (2013) [10]
14	Mealybug Stress Index-2 (MSI-2)	(R_550_ + R_768_) − (R_674_ + R_1454_)/(R_1454_ + R_674_) + (R_550_ + R_768_)	Prabhakar et al. (2013) [10]
15	Mealybug Stress Index-3 (MSI-3)	(R_550_ − R_674_)/(R_550_ + R_674_)	Prabhakar et al. (2013) [10]
16	Plant Pigment Ratio (PPR)	(R_550_-R_450_)/(R_550_ + R_450_)	Metternicht (2003) [34]
17	Aphid Stress Index 1(ASI-1)	(R_666_-R_1462_)/(R_666_ + R_1462_)	Present study
18	Aphid Stress Index 2(ASI-2)	(R_1908_/ R_1964_)	Present study

R, reflectance at corresponding wavelength (nm) depicted as subscript.

**Table 4:** Comparison of leaf reflectance between healthy (Grade 0) and aphid infested cotton plants (Grades 1–4) at five broad-band regions of electromagnetic spectrum.

Parameter	Spectral Region				
	Blue (450–520 nm)	Green (521–600 nm)	Red (630–690 nm)	NIR (760–900 nm)	SWIR (1550–1750 nm)
Grade 0					
Mean reflectance values	0.0778 ± 0.0098	0.1461 ± 0.0184	0.0862 ± 0.0105	0.5912 ± 0.0147	0.3235 ± 0.0052
Grade 1					
Mean reflectance values	0.0765 ± 0.0160	0.1393 ± 0.0168	0.0811 ± 0.0132	0.5505 ± 0.0141	0.3200 ± 0.0171
Pr > |t|	<0.00001 **	<0.0001 **	<0.0001 **	<0.0001 **	<0.0001 **
Grade 2					
Mean reflectance values	0.0620 ± 0.0097	0.1135 ± 0.0113	0.0648 ± 0.0078	0.5003 ± 0.0074	0.3020 ± 0.0126
Pr > |t|	<0.0001 **	<0.0001 **	<0.0001 **	<0.0001 **	<0.0001 **
Grade 3					
Mean reflectance values	0.0545 ± 0.0063	0.1020 ± 0.0134	0.0575 ± 0.0061	0.4556 ± 0.0107	0.2852 ± 0.0092
Pr > |t|	<0.0001 **	<0.0001 **	<0.0001 **	<0.0001 **	<0.0001 **
Grade 4					
Mean reflectance values	0.0503 ± 0.0004	0.0982 ± 0.0070	0.0526 ± 0.0033	0.4313 ± 0.0137	0.2712 ± 0.0009
Pr > |t|	<0.0001 **	<0.0001 **	<0.0001 **	<0.0001 **	<0.0001 **

** Highly significant (at <0.01%).

**Table 5:** Mean chlorophyll concentration and relative water content (RWC) in cotton plants with varying levels of aphid severity.

Damage Severity	Chl a (μg/cm^2^)	Chl b (μg/cm^2^)	Chl a + b (μg/cm^2^)	RWC (%)
Grade 0	46.67 a	14.00 a	60.67 a	79.49 a
CV (%)	3.27	7.14	0.95	1.06
Grade 1	40.00 b	11.67 b	51.67 b	78.03 a
CV (%)	5.00	4.95	4.03	1.81
Grade 2	31.67 c	8.67 c	40.33 c	70.89 b
CV (%)	4.82	6.66	2.86	1.24
Grade 3	24.00 d	5.67 d	29.67 d	68.50 c
CV (%)	4.17	20.38	7.02	1.12
Grade 4	17.67 e	3.33 e	21.00 e	63.31 d
CV (%)	6.53	45.83	12.59	1.58
LSD	**	**	**	**

CV, Coefficient of variation; LSD, Least significant difference; Values followed by a different small letter within the same column mean significantly different at 0.05 probability level. ** significant differences at 0.01 probability level respectively.

**Table 6:** Performance of different hyperspectral vegetation indices in linear regression model for assessing cotton aphid damage severity.

	Spectral Vegetation Index	R^2^	Slope	RMSE
1	Simple Ratio (SR)	0.62	25.365	0.72
2	Normalized Difference Vegetation Index (NDVI)	0.07	8.69	1.12
3	Disease Water Stress Index 2 (DWSI-2)	0.43	3.1765	0.88
4	Aphid index (AI)	0.25	−9.8368	1.00
5	Damage sensitive Spectral Index-2 (DSSI 2)	0.12	0.1429	1.09
6	Chlorophyll Index (CI)	0.16	17.321	1.06
7	Chl Stress Index 1 (Chl SI-1)	0.08	3.1007	1.11
8	Chl Stress Index 2 (Chl SI-2)	0.08	4.1117	1.16
9	Chl Stress Index 3 (Chl SI-3)	0.08	−14.106	1.11
10	Leaf Hopper Index (LHI)	0.40	7.1082	0.89
11	Nitrogen Stress Index 1 (NSI-1)	0.02	3.2615	1.15
12	Nitrogen Stress Index 2 (NSI-2)	0.05	−0.9661	1.13
13	Mealybug Stress Index-1 (MSI-1)	0.54	−7.8639	0.78
14	Mealybug Stress Index-2 (MSI-2)	0.57	−5.1301	0.76
15	Mealybug Stress Index-3 (MSI-3)	0.02	2.8806	1.15
16	Plant Pigment Ratio (PPR)	0.02	−2.5944	1.15
17	Aphid Stress Index 1 (ASI-1)	0.81	10.993	0.50
18	Aphid Stress Index 2 (ASI-2)	0.80	25.987	0.51
